# Root canal morphology of maxillary second premolars in an Indian population

**DOI:** 10.4103/0972-0707.71648

**Published:** 2010

**Authors:** Udayakumar Jayasimha Raj, Sumitha Mylswamy

**Affiliations:** Department of Conservative Dentistry and Endodontics, Meenakshi Ammal Dental College, Alapakkam Main Road, Maduravoyal, Chennai, Tamil Nadu, India

**Keywords:** Apical delta, decalcification and staining, maxillary second premolar, root canal morphology, Vertucci’s classification

## Abstract

**Introduction:**

The purpose of this study was to investigate root canal morphology of maxillary second premolars in an Indian population.

**Materials and Methods:**

Two hundred maxillary second premolars were collected, stained, decalcified, and cleared. Cleared teeth were examined in a steromicroscope under 7.5× magnification and the following observations were made: (1) length of the teeth, (2) number of root canals, (3) root canal configuration by Vertucci’s classification, (4)number of isthmi between the canals, (5) frequency of apical deltas.

**Results:**

Of the two hundred maxillary second premolars, 64.1% had one root canal at the apex and 35.4 % had two root canals at the apex. The average length of the teeth was 21.5 mm. Concerning the canal morphology, 33.6% of the teeth exhibited Vertucci type II configuration followed by type IV pattern (31.1%); 29.2% of the teeth possessed type I pattern. An additional canal configuration type XIX was found in one tooth. Isthmi and apical deltas was found in 19% and 14% of the cases, respectively.

**Conclusion:**

The root canal morphology of Maxillary second premolars can be complex and requires careful evaluation prior to endodontic therapy.

## INTRODUCTION

The success of root canal therapy depends on a thorough knowledge of the root and root canal morphology so as to locate all existing canals and properly clean, shape, and obturate the root canal space three-dimensionally.[1] Studies on the internal and external anatomy of teeth have shown that anatomic variations can occur in all groups of teeth and can be extremely complex.[2,3] Numerous factors contribute to the variations found in the root canal studies including ethnicity,[4,5] age,[6] gender,[7] and study design (*in vitro* versus *in vivo*).[8] The maxillary second premolars are among the most difficult teeth to be treated endodontically. This could be due to many factors namely the number of roots, the number of canals, the direction and longitudinal depressions of the roots, the various pulp cavity configurations, and the difficulties in visualizing the apical limit by radiographs.[9] Many studies have investigated the root canal morphology of maxillary second premolar teeth and reported significant variations. However, these studies were mainly performed on teeth of North American,[3] Turkish,[7] and Chinese populations.[10]

There are no published reports on the root canal anatomy of maxillary second premolars in Indian population till date.

Hence, this study was undertaken to investigate the root canal anatomy of maxillary second premolar in an Indian population using Vertucci classification and to compare these findings with the published reports of different population.

## MATERIALS AND METHODS

Two hundred extracted human adult maxillary second premolar teeth from an Indian population were collected. Teeth with fracture, incompletely formed roots, metallic restorations, and deep caries were not included. The teeth were stored in 10% formalin (Western India Chemical, Udupi District, Karnataka, India). Calculus and stains were removed using an ultrasonic scaler. The length of the teeth was measured using vernier caliper from the tip of the crown to the apex of the root. In case of a curved root, tangents were drawn to the curved portions of the tooth. The lengths were then measured by connecting the points of tangency. Access cavities were prepared using No. 2 round bur and the pulp tissue was dissolved by immersing the teeth in 2.5% sodium hypochlorite (Prime Dental Products Pvt. Ltd., Mumbai, India) for 12 hours, followed by 20 minutes immersion in an ultrasonic bath. The teeth were then rinsed under running tap water for 2 hours and dried overnight.

A syringe with a gauge 27 needle was used to inject the India Ink (Emichem Pvt. Ltd., Kolkata, India) into the root canal spaces coronally, assisted by vacuum suction apically. The teeth were air dried and decalcified in 5% nitric acid (George Chem, Vellore, Tamil Nadu, India) for 4–5 days. The acid solution was changed daily and the end point of decalcification was determined by periodic radiographs. The teeth were washed under running water to remove traces of nitric acid, dried and dehydrated using increasing concentrations of ethanol (70%, 95%, 100%) (Leonid Chemicals Pvt. Ltd., Bangalore, Karnataka, India) for 24 hours. Finally the teeth were rendered transparent by immersing in methyl salicylate (Jain General Traders, Chennai, Tamil Nadu, India). The cleared teeth were examined under stereomicroscope under 7.5× magnification.

The canal configurations were categorized into the first seven types of Vertucci’s classification (1984) as follows:

1. type I. A single canal present from the pulp chamber to the apex;
2. type II. Two separate canals leave the pulp chamber and join short of the apex to form one canal;
3. type III. One canal leaves the pulp chamber, divides into two within the root, and then merges to exit in one canal;
4. type IV. Two separate and distinct canals are present from the pulp chamber to the apex;
5. type V. Single canal leaves the pulp chamber but divides into two separate canals with two separate apical foramina;
6. type VI. Two separate canals leave the pulp chamber but join at the midpoint and divides again into two separate canals with two separate apical foramina; and
7. type VII. One canal leaves the pulp chamber, divides and rejoins within the canal, and finally redivides into two distinct canals short of the apex.

## RESULTS

**Figure 1:**
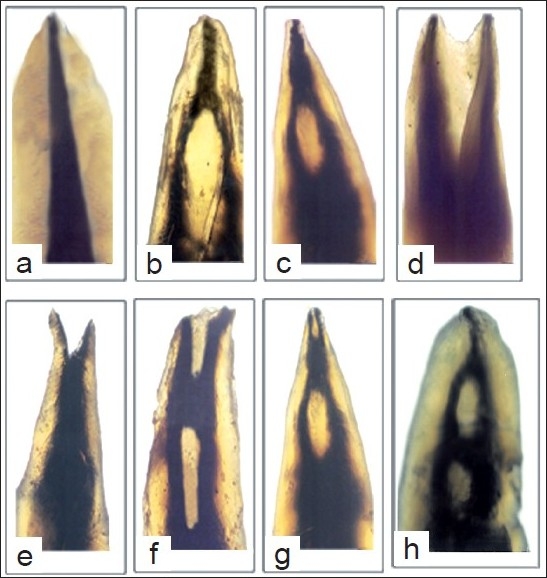
Canal configurations observed in this study. a – type I, b – type II, c – type III, d – type IV, e – type V, f – type VI, g – type VII, and h – type XIX

**Figure 2:**
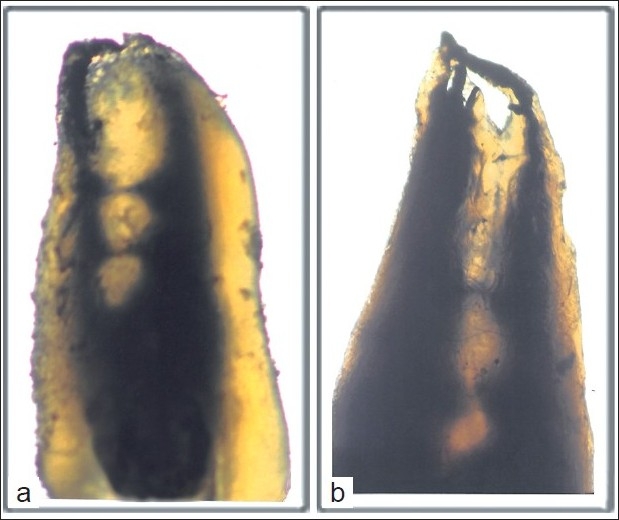
a) Isthmi in the middle third of the root. b) The apical delta.

Of the 200 studied maxillary second premolars, 64.1% had one root canal at the apex and 35.4% had two root canals at the apex. The average length of these teeth was 21.5 mm, ranging from 15.5 to 28 mm. In this study, variable root canal configurations were found in maxillary second premolars. Type II configuration was most prevalent (33.6%) followed by type IV (31.1%), type I (29.2%), type V (2.1%), type III (1.3%), type VI (1.2%), and type VII (1%) [Figure 1a–g]. One teeth showed an additional configuration, Sert and Bayirli’s type XIX (2-1-2-1): two separate canals leave the pulp chamber, join at the midpoint and divides again into two separate canals and rejoins to form one canal at the site of exiting [Figure 1h]. Isthmi [Figure 2a] and apical deltas [Figure 2b] were found in 19% and 14% of the cases examined.

## DISCUSSION

This study examined the root canal morphology of maxillary second premolar in an Indian population. Studies on root canal morphology have been conducted by various methods like radiography,[11] decalcification and clearing,[3–5] direct observation with microscope,[12] sectioning and macroscopic observation,[13] and computer tomography.[14] Although various techniques have been used in these studies evaluating canal morphology, it has been reported that the most detailed information can be obtained ex vivo by demineralization and staining.[3] This technique also makes canal negotiation with instruments unnecessary, thereby maintaining the original form and relation of canals and provides a three-dimensional view of root canal.[3,4] The process of changing the tooth into a transparent object involves many physical and chemical changes. The inorganic constituents of the tooth are first dissolved by decalcification and further water, air, and lipid components are removed by dehydration and by subsequent immersion in the clearing agents.[15] The decalcifying agent 5% nitric acid is rapid in action, causes little damage to tissue if the time of decalcification is rapidly controlled.[16] After fixation in aqueous solutions, tooth tissue needs to be dehydrated slowly in order to prevent high degree of shrinkage due to the rapid removal of water.[16] When the dehydrating agent has been entirely replaced by methyl salicylate, the tissue has a transparent appearance, as the clearing agent increases the refractive index of the tooth.[16] The use of stereomicroscope for viewing the root canal pattern resulted in higher accuracy and magnification when compared to the magnifying glasses that were used in the previous studies.

The number of root canals in the maxillary second premolars shows wide variation. Table 1 compares the results of the present study with other studies of maxillary second premolars. In this study, it was found that 35.4% of maxillary second premolars had two root canals at the apex. This is lower than several earlier studies.[10,17–20] but considerably higher than that reported by Pineda and Kuttler[2], Vertucci[3] and Caliskan *et al*[21] An interesting observation is that the percentage of maxillary second premolars with a single canalat the apex was 64.1%, which shows a higher percentage than studies conducted by various authors[10,17–20,22] but lower than studies of Pineda and Kuttler[2], Vertucci[3],Pecora *et al*[9] and Caliskan *et al*[21] The average length of the teeth in this study was similar to the findings of Pecora *et al*[9] The most prevalent canal pattern in this study was type II occurring in 33.6% of maxillary second premolars followed by type IV, type I, type V, type III, type VI, and type VII. It was found that 29.2% of the teeth had only one canal. This percentage is much lower than earlier studies,[3,7,18] but slightly higher than in the Chinese population.[10] Of the special interest was the single maxillary second premolar with Sert and Bayirli type XIX configuration, which makes the treatment of this teeth challenging. There are a number of published case reports indicating the presence of three canals in maxillary second premolars.[23,24] But in the present study, none of the samples had three canals.

**Table 1:** Percent of root canal of maxillary second premolars in various studies

Author	Year	No of canals			
		No of teeth	One canal at apex (%)	Two canals at apex (%)	Three canals at apex (%)
Pineda and Kuttler[2]	1972	282	81.8	18.2	0
Vertucci[11]	1974	200	75	24	1
Bellizzi and Hartwell[12]	1985	630	40.3	58.6	1.1
Pecora *et al*.[9]	1992	300	67.3	32.4	0.3
Caliskan *et al*.[13]	1995	100	72	28	0
Chima[14]	1994	26 females	30	73	0
		20 males	30	70	0
Kartal *et al*.[11]	1997	300	55	44.4	0.6
Sert and Bayirli[7]	2004	100 females	75	24	1
		100 males	49	48	3
Khurram *et al*.[15]	2005	57 females	47	53	0
		43 males	37	63	0
Rozylo *et al*.[16]	2008	56	14.7	85.3	0
Weng *et al*.[10]	2009	65	27.7	72.3	0
Present study[*]	2010	200	64.1	35.4	0

* Type XIX (Sert and Bayirli) – 0.5%

The data for maxillary second premolar canal configuration of this study compared with other studies are shown in Table 2. The differences between these morphological studies may be related to the variations of examination methods, classification systems, sample sizes,[8] and racial and genetic factors.[25]

**Table 2:** Root canal morphology of maxillary second premolars in various studies

Reference	No. of teeth	Type of study	Canal Types							
			I (%)	II (%)	III (%)	IV (%)	V (%)	VI (%)	VII (%)	VIII (%)
Vertucci[3] (USA) 100 Clearing	100	Clearing	48	22	5	11	6	5	2	1
Caliskan *et al*.[13] (Turkey)	100	Clearing	44	22	6	12	6	6	4	-
Kartal *et al*.[11]	300	Clearing	48.6	6.3	-	37.99	4	0.6	-	-
Sert and Bayirili[7] (Turkey)	200	Clearing	32	20	10	25.5	6	1.5	3	1.5
Weng *et al*.[10] (China)	100	Clearing	27.7	36.9	-	33.8	-	1.6	-	-
Present study (2010) (India)	200	Clearing	29.2	33.6	1.3	31.1	2.1	1.2	1	-

The preparation and obturation of types I and IV canal system are relatively straightforward because each of the canals in these configurations is separate and distinct between orifice and apex. However, types II, III, V, VI, and VII systems are different because there are areas in the root in which the two canals join and share the pulp space, and others in which the canals are separate. This requires an individualized procedure for location of the furcation area as well as the precise position of the root canal orifices, preparation, and filling in each of these conditions to obtain the most desirable results.[8] If this cannot be achieved, the negotiation of the entire root canal system is questionable, and the long-term prognosis for the tooth may become extremely poor.

Cambruzzi and Marshall[26] called an intercanal connection or transverse anastomosis as “isthmus” and stressed the importance of preparing and obturating it during surgery. An isthmus is a narrow, ribbon-shaped communication between two root canals that contains pulp or pulpally derived tissue. It can also function as a bacterial reservoir.[3] Any root that contains two or more root canals has the potential to contain an isthmus.[3]

In this study, isthmi were observed in 19% of the cases, which is lower than that reported by Vertucci[3] and Weng *et al*.[10] [Table 3], but consistent with the findings of Sert and Bayirili[7] and Caliskan *et al*.,[21] Apical deltas were observed in 14% of maxillary second premolars, which is similar to the findings of Vertucci[3] and Kartal *et al*.[18] but lower than that reported by Caliskan *et al*.,[2,13] Sert and Bayirli,[7] and Weng *et al*.[10] The apical delta is difficult to debride and may predispose to endodontic failure.

**Table 3:** Number and percentage of Isthmi and apical deltas

Reference	Number of teeth	Transverse anastomosis (%)	Apical deltas (%)
Vertucci[3]	100	30.8	15.1
Caliskan *et al*.[13]	100	20	26
Kartal *et al*.[11]	300	37	15
Sert and Bayirili[7]	200	20.5	26.5
Weng *et al*.[10]	100	65	43.8
Present study (2010)	200	19	14

## CONCLUSION

The root canal morphology of the maxillary second premolar in Indians shows a higher incidence of type II configuration (33.6%). The finding of additional type, namely type XIX (2-1-2-1), is rare but should be kept in mind when performing endodontic therapy for these teeth. The outcomes of nonsurgical and surgical endodontic procedures are influenced by highly variable anatomic structures. Therefore clinicians ought to be aware of complex root canal structures, cross-sectional dimensions, and iatrogenic alterations of canal anatomy.
